# A study on the relationship between role stress and job burnout among community mental health workers: using psychological capital as a mediating variable

**DOI:** 10.1186/s40359-026-04532-3

**Published:** 2026-04-25

**Authors:** Hsueh-Kan Lu, Hui-Chuang Chu, Yin-Che Chen

**Affiliations:** 1 Taipei Municipal Chenggong High School, Taipei, Taiwan; 2 Department of Educational Psychology and Counselling, National Tsing Hua University, Hsinchu, Taiwan

**Keywords:** Community mental health care system personnel, Role stress, Job burnout, Psychological capital

## Abstract

**Research purpose:**

Mental health care plans and services worldwide are increasingly transitioning toward community-based models (Cochrane Database Syst Rev 1:CD00790, 2017; Innovative care for chronic conditions in building blocks for action, 2002; J Nurs 62:5–11, 2015). In our country, there was a significant burden on and instability in the manpower of personnel in the community mental health care system, as compared to patients. However, there was a lack of research on the role of psychological capital as a mediator between role stress and job burnout among personnel in the community mental health care system. This study aims to explore the interrelationships among role stress, psychological capital, and job burnout among community mental health care personnel. Specifically, we investigate the predictive effect of role stress on job burnout and examine the mediating role of psychological capital within this relationship.

**Research method:**

The subjects of this study were personnel in the community mental health care system, and an online questionnaire was used. A total of 313 questionnaires were collected, with 262 valid responses. Data analysis and interpretation were conducted using SPSS and SPSS AMOS for descriptive statistics, structural equation modeling, and bootstrapping.

**Research results:**

The research results were presented as follows: (1) The level of role stress among community mental health care system personnel was moderate to low (*M* = 2.78, *SD* = 0.78); (2) The level of job burnout among community mental health care system personnel was moderate to low (*M* = 2.91, *SD* = 0.842); (3) The level of psychological capital among community mental health care system personnel was moderate to high (*M* = 3.48, *SD* = 0.737); (4) Role stress significantly positively predicted job burnout (*β* = .379, *t* = 4.632, *p <* .001); (5) Psychological capital among community mental health care system personnel partially mediated the relationship between role stress and job burnout (*β* = .227, *p* = .001).

**Discussion and conclusion:**

Based on these findings, recommendations were proposed for the cultivation and management of community mental health care system personnel, such as organizing meetings to reduce role conflicts, planning and managing manpower to alleviate role overload, and establishing mechanisms for personnel training to enhance psychological capital.

## Background

Mental health care has been gradually shifting from the traditional hospital-centered and treatment-oriented approach to a community-based model that takes into account the physical and psychological conditions of patients with mental illness as well as their future adaptation to daily life [1], with the goal of encouraging patients to return to the community [2]. Similarly, global mental health care policies have shown a trend toward deinstitutionalization, demedicalization, and recovery-oriented community care. For example, mental health policies in Australia are primarily implemented through collaborations between local community mental health centers and hospitals, supported by Crisis Assessment and Treatment Services (CATS), Mobile Support Teams (MST), and Continuing Care Teams (CCT). These teams not only provide psychiatric, psychological, social, functional, and family assessments but also connect patients with various rehabilitation and functional support resources [3]. Meanwhile, in the US, an intensive case management (ICM) program for mental health care has been developed based on the Assertive Community Treatment (ACT) and Case Management (CM) models. The ICM program emphasizes a small caseload that entails a case manager-to-client ratio of less than 1:20, along with the delivery of high-frequency, intensive community-based services [4]. In Taiwan, community mental health care is mainly implemented through the establishment of a community mental health inspection system, which integrates the efforts of public health nurses at local health centers and mental health social workers to improve community-based care for patients reintegrating into the community. Relevant personnel were recruited by the Ministry of Health and Welfare [5] (MOHW) using a project-based subsidy mechanism. Although the system was designed to achieve a targeted caseload ratio of 1:30 based on international standards, the actual recruitment figures fell short. Specifically, only 216 personnel were hired that year, of whom merely half served as community mental health inspectors. Meanwhile, the total number of registered cases reached 34,861 in the same year, resulting in an average caseload ratio of approximately 1:323. Compared to a ratio of 1:10 reported by the MST team in Australia, 1:20 by the CCT team in Australia, and 1:20 by the ICM program in the US, the local caseload ratio reveals that mental health workers in Taiwan experience substantial work overload, making it difficult to deliver adequate and high-quality services to clients and their families [4, 6–9]. Moreover, Numerous studies in Taiwan have pointed out that personnel in the community mental health care system frequently experience work overload due to factors such as excessive caseloads, extensive travel required by large service areas, time-pressured tasks, communication barriers between workers and supervisors, insufficient work experience, or a lack of adequate organizational and managerial support. These factors lead to high turnover rates and frequent changes in frontline staff. Such personnel mobility and turnover further impose pressure on senior staff, who must undertake the responsibility of training newcomers. Additionally, research indicates that care system personnel face challenges such as insufficient referral resources, difficulties in the referral process, and rejection by clients [10–12]. From the perspective of the Job Demands–Resources (JD-R) model, these unfavorable working conditions can be conceptualized as excessive job demands that require sustained physical and psychological effort, thereby increasing the likelihood of energy depletion and subsequent burnout [13]. Drawing on Conservation of Resources (COR) theory, hese persistent role-related stressors of personal accomplishmentmay initiate a resource loss spiral, in which individuals gradually lose their psychological and emotional resources, ultimately leading to emotional exhaustion and burnout [14]. According to COR theory, individuals strive to obtain, retain, and protect their valued resources, and psychological strain occurs when these resources are threatened or depleted.

Integrating the JD-R and COR perspectives provides a clearer explanatory mechanism: job demands such as role stress (e.g., role conflict, role ambiguity, and role overload) contribute to resource depletion, whereas personal resources play a critical role in buffering this process. In this regard, psychological capital (PsyCap), characterized by hope, self-efficacy, resilience, and optimism, can be conceptualized as a key personal resource that helps individuals cope with job demands, maintain adaptive functioning, and mitigate the negative impact of stress on burnout outcomes.

While Cao et al. [6] indicated that individuals with higher levels of positive psychological capital were more capable of preventing and mitigating job burnout, while Cheng and Sung [15] observed that both Western and Taiwanese studies on psychological capital have seldom examined psychological capital as a mediating or moderating variable in a systematic manner. This gap suggests the need for a more theoretically grounded investigation into the mechanism through which PsyCap operates within high-demand work contexts.

Therefore, this study aimed to investigate the relationship between role stress (specifically role conflict, role ambiguity, and role overload) and job burnout among community mental health workers, and further explore whether PsyCap plays a mediating role in this relationship. By doing so, this study not only clarifies the resource-based mechanism underlying burnout but also contributes to a more integrated application of the JD-R and COR frameworks in community mental health settings, thereby augmenting the knowledge about the multifaceted nature of their job roles and providing evidence-based recommendations.

### Role stress

Role stress refers to a state in which an individual is unable to effectively perform a specific role. Agrawal and Chahar [16] conceptualized role stress as conflicts between the self, the role under question, and other roles that the person occupies. Chen and Cheng [17] indicated that social workers experience role stress when confronted with inconsistent expectations and excessive workloads. Maor and Hemi [18] defined role stress as the stress employees experience because of the roles they occupy within their organizations. From a resource perspective, some scholars suggest that role stress arises when individuals experience psychological tension due to the belief or anticipation that the behaviors required of them place greater demands on them than their available resources can meet [19]. Role stress reflects the challenges and difficulties individuals experience in performing their roles. It is influenced by not only personal capabilities but also organizational and interpersonal dynamics, which collectively influence the individual’s subjective experience and psychological responses within their role.

Previous studies have typically divided role stress into three dimensions: role ambiguity, role conflict, and role overload [20–22]. Role ambiguity occurs when individuals lack explicit authorization regarding their rights and responsibilities, making it difficult to comprehend the expectations associated with their role and, consequently, to exhibit appropriate role behaviors. Role ambiguity is distinct from role conflict [23–25], as it refers to incompatibility or competition among expectations associated with different roles [24]. Roles are formed through behavioral expectations derived from social structures [15, 26], whereas role conflict arises when such behaviors are incompatible with the expectations of other stakeholders. Role overload occurs when individuals assume excessive responsibilities and fail to handle them owing to capacity and time limitations. It includes both the “quantitative” aspect, that is, workload, and the “qualitative” aspect, that is, task difficulty [27, 28.

Role stress may affect multiple aspects of an individual’s performance, including physical and psychological condition, self-efficacy, job burnout, job satisfaction, retention intention, withdrawal behavior, turnover intention, and well-being [17, 19, 20, 29, 30]. In addition to its impact on individuals, role stress can also influence organizations, for example, by increasing workplace withdrawal behaviors [31, 32]. Generally, role stress exerts multiple negative effects, encompassing physiological and psychological dimensions in both individuals and organizations, ultimately leading to increased organizational burdens and detriments.

### Job burnout

Job burnout, also referred to as professional burnout, occupational fatigue, or occupational exhaustion, is particularly prevalent among helping professions such as caregiving, education, and healthcare [33–35]. It significantly affects physical and mental well-being as well as work efficiency, and may lead to early retirement or resignation [36–38]. Kristensen et al. [39] defined job burnout as a state of physical and psychological fatigue and exhaustion that may derive from factors such as family issues, personal illness, social relationships, work organization, and interactions with service recipients. Job burnout reflects individuals’ psychological and physiological responses to prolonged stress, yielding negative perceptions and attitudes toward their work, interpersonal relationships, and overall life circumstances. As a mental symptom, it manifests as emotional exhaustion, depersonalization, and a diminished sense of personal accomplishment [40–42, 100].

According to Maslach et al. [41], job burnout can be conceptualized in three dimensions: emotional exhaustion, cynicism, and a sense of reduced personal accomplishment. Conversely, Kristensen et al. [39] focused on the concept of burnout itself, proposing three dimensions for burnout evaluation: personal burnout, work-related burnout, and client-related burnout. Personal burnout refers to a general feeling of exhaustion and depletion that stems from not only work but also social interactions, interpersonal relationships, or illness. Work-related burnout focuses on exhaustion arising from work tasks. Client-related burnout specifically measures the fatigue experienced during interaction with clients or service recipients. Job burnout may induce physical, psychological, and emotional issues [43–45]. Furthermore, burnout undermines an individual’s creativity and may result in substance abuse behaviors such as alcoholism or smoking. It may also manifest as psychological symptoms such as anxiety, insomnia, and even an intention to leave the profession because of job dissatisfaction [46]. Burnout can induce apathy, detachment, and demotivation among workers toward their work [47]. Additionally, it is associated with poor interpersonal relationships, depression, suicidal tendencies, and even reduced life expectancy [48]. Studies have recommended that administrative bodies or organizations should regularly assess employees’ mental health status [49, 50]. Researchers posit that job burnout is not only related to occupational stress, workload, and job satisfaction but also to environmental factors such as organizational climate and workplace violence. These factors, in turn, may harm organizations by damaging their reputation and mitigating staff retention rates [51–53].

### Psychological capital

According to Luthans et al. [54], psychological capital comprises self-efficacy/confidence, optimism, hope, and resilience, and these are intercorrelated, rather than independent or singular dimensions. Notably, some studies have proposed modifications to the components of psychological capital based on cultural differences between Eastern and Western contexts, such as including serenity as an additional component and regarding hope and self-efficacy as a unified dimension [55, 56]. However, other researchers continue to affirm that the four dimensions of psychological capital are correlated [57, 58].

Both domestic and international studies suggest that psychological capital can mitigate job burnout [14], predict job satisfaction [59], and enhance organizational citizenship behavior [38, 60–62] ). Additionally, psychological capital not only mediates the relationship between a supportive organizational climate and employee performance [63], but also moderates factors such as work stress, job security, and role stress, thereby influencing outcomes related to job burnout and psychological well-being [31, 64, 65].

### Role stress and job burnout

Numerous studies have indicated that when individuals experience role stress at work, the resulting imbalance between job demands and available resources can induce job burnout [66, 67]. Furthermore, a positive correlation has been established between role stress and job burnout [29, 68–70]. Specifically, role stress exerts a direct impact on employees’ job burnout, which, in turn, affects other dimensions such as ethical decision-making and work quality [71]. Some studies suggest that the relationship between role stress and job burnout can be moderated by factors such as emotional labor. Based on these findings, Hypothesis 1 is proposed, that is, role stress among community mental health workers has a predictive effect on their job burnout.

### Role stress, psychological capital, and job burnout

As a mediating variable, psychological capital can mitigate the adverse effects of organizational issues, such as job insecurity [72], worlplace bullying [73] and job burnout [74]. Ttheoretically, this mediation is grounded in the integration of the Job Demands–Resources (JD-R) model and the Conservation of Resources (COR) theory [75].

In the Job Demand-Resource (JD-R) model proposed by Demerouti et al. [13], job demands refer to aspects of work that require sustained physical or psychological effort to meet physiological, interpersonal, and organizational requirements, whereas personal resources—such as psychological capital—are defined as the internal assets that enable individuals to successfully control and impact their environment. Within this framework, role stress (specifically role conflict, role ambiguity, and role overload) is conceptualized as a chronic job demand that triggers a "health impairment process," leading to energy depletion and eventual burnout. Conversely, COR theory [76] posits that individuals experience stress when their valued resources are threatened or lost. From this perspective, PsyCap functions as a "resource caravan" [75] that provides a protective buffer against the resource depletion caused by persistent role stressors.

The synergy between JD-R and COR theories provides a robust mechanism to explain the mediation process. Specifically, while role stress accelerates the loss of emotional and cognitive energy, high levels of PsyCap—comprising hope, efficacy, resilience, and optimism—allow workers to avoid this "energy depletion" route [14, 31]. By integrating these frameworks, it is argued that PsyCap not only directly counteracts negative reactions to work characteristics through self-regulation [77] but also facilitates a restorative process that maintains psychological well-being despite high job demands.

The theoretical foundation for this mediation was rooted in the integration of the Conservation of Resources (COR) theory and the Job Demands–Resources (JD-R) model. In this view, psychological capital acts as a "resource caravan" that protects individuals from the resource depletion caused by chronic stressors [75]. Incorporating a granular perspective, the various components of role stress are conceptualized as distinct job demands that initiate a health impairment process. While quantitative overload depletes energy through physical and time pressure, role conflict and ambiguity drain resources through cognitive dissonance and emotional labor; collectively, these demands accelerate the path toward exhaustion.

By conceptualizing role stress (e.g., role conflict, role ambiguity, and role overload) as a primary job demand and PsyCap as a personal resource, researchers suggest that individuals with higher PsyCap can avoid the **"**energy depletion" route to resource exhaustion [14, 31, 78, 79]. Specifically, Demerouti [77] expanded the JD-R theory by proposing that personal resources directly counteract negative reactions to work characteristics, thereby maximizing positive effects through self-regulation.

Furthermore, when examining the granular components of role stress, including role conflict and role ambiguity, psychological capital allows workers to cognitively reframe these stressors as challenges rather than insurmountable threats [77]. For instance, in a study involving university professors, Heng et al. [74] examined the effect of teaching–research conflict on job burnout and identified that psychological capital played a mediating role in this relationship. Given that teaching–research conflict experienced by university professors is a form of role conflicts, it can be inferred that higher levels of hope, efficacy, resilience, and optimism (the core components of PsyCap). This granular approach underscores that PsyCap serves as a versatile psychological buffer tailored to the unique pressures of each role stress dimension weaken the link between conflicting role expectations and emotional exhaustion.

Alternatively, Bakker and de Vries [80] indicated that key personal resources, such as proactive personality, can help employees effectively identify and self-regulate fatigue. Specifically, individuals with higher psychological capital are more capable of recognizing and managing their own fatigue levels. In summary, as a personal resource in work, psychological capital enables employees to cope with specific role stress components.

In line with the JD-R model, job demands (role stress) are expected to increase burnout through a health impairment process, whereas personal resources (psychological capital) can buffer this process and promote resilience [81, 82]. In other words, psychological capital helps workers maintain positive emotions and resilience while preventing exhaustion under role stress. Furthermore, previous studies indicate that psychological capital effectively predicts job burnout among employees [57, 83, 84].

Based on the integrated JD-R and COR framework, this study proposes a theoretically grounded mediation model in which role stress depletes individual resources, psychological capital functions as a buffering and restorative resource, and burnout represents the outcome of prolonged resource imbalance. Therefore, this study posits that the relationship between role stress and job burnout is influenced by the individual’s level of psychological capital. On this basis, Hypothesis 2 is proposed: psychological capital mediates the relationship between role stress and job burnout among community mental health workers.

Integrating these perspectives, role stress is expected to negatively influence psychological capital, which in turn affects burnout levels, forming an indirect pathway through resource depletion and buffering mechanisms.

Accordingly, the following hypotheses are proposed:

- H1: Role stress positively predicts job burnout among community mental health workers.
- H2: Psychological capital mediates the relationship between role stress and job burnout.

## Methods

### Research subjects

Personnel defined in the “Implementation Guidelines for the Integrated Mental Health Program ” subsidized by the MOHW [9] and implemented by the health bureaus of municipalities and county (city) governments in Taiwan were recruited as research subjects. A voluntary online questionnaire survey was conducted nationwide, targeting community mental health workers whose responsibilities included suicide prevention and mental illness-related services. Given that all variables were collected through self-report measures from the same participants at a single point in time, common method bias (CMB) may pose a potential concern. To address this issue, Harman’s single-factor test was conducted. The results indicated that the first unrotated factor accounted for less than 40% of the total variance, suggesting that no single factor dominated the variance. Therefore, CMB was not considered a serious threat in this study. Data collection spanned from January to April 2024 using online questionnaires and convenience sampling. In addition, procedural remedies such as assuring anonymity and reducing evaluation apprehension were applied to minimize potential method bias.

The questionnaire consisted of four sections. The first section covered demographic information, including sex, age, marital status, educational level, educational background, professional certification, type of the current employing organization, years of service in the current employing organization, and average weekly working hours. The remaining three sections adopted three standardized scales developed by researchers. According to Podsakoff and Organ [85], self-report surveys are subject to the risk of common method variance (CMV). In this study, Harman’s single-factor test was employed for post hoc detection of CMV. A total of 313 questionnaires were collected. After excluding 51 invalid responses, 262 valid questionnaires were retained, representing an effective response rate of 83.7%.

### Research instruments

#### Role stress scale

This study employed the “Role Stress Scale” developed by Chen and Cheng [17] comprising19 items scored on a 5-point Likert scale. A total of four dimensions were covered: role ambiguity (four items), role conflict (seven items), role overload (quantitative) (four items), and role overload (qualitative) (four items). Regarding reliability, the overall Cronbach’s α for the original scale was 0.873, while the α coefficients for each subscale ranged from 0.747 to 0.955. Validity was examined via factor analysis, with the four factors cumulatively explaining approximately 76.517% of the total variance. In the present study, item analysis and factor analysis led to the removal of items 5, 6, 7, 9, 12, and 13 to ensure optimal construct validity. For the refined scale, the Cronbach’s α for the subscales ranged from 0.827 to 0.888, with an overall α of 0.920. To further verify the measurement model's construct validity. Confirmatory factor analysis (CFA) was further conducted to validate the measurement structure. After adjusting for residual covariances between certain items (e.g., items 10 & 16), the results showed that all standardized factor loadings were significant (ranging from 0.605 to 0.927). The results indicated that all standardized factor loadings exceeded 0.60, demonstrating acceptable indicator reliability. Composite reliability (CR = 0.865) values for all subdimensions exceeded the recommended threshold of 0.70, and the average variance extracted (AVE = 0.622) values were above 0.50, supporting satisfactory convergent validity. The modified model demonstrated good fit indices: χ2/df = 2.640, GFI = 0.903, AGFI = 0.854, and RMSEA = 0.079. Furthermore, discriminant validity was confirmed as the confidence intervals of the correlations among latent constructs did not include 1. These findings suggest that the Role Stress Scale demonstrates adequate construct validity and reliability in the context of community mental health workers.

#### Job burnout scale

This study applied the “Chinese Version of the Workplace Fatigue Scale” developed by Yeh et al. [45] comprising 21 items that encompassed four dimensions: personal burnout (five items), work-related burnout (five items), client-related burnout (six items), and overcommitment to work (five items). Each item was rated on a 5-point Likert scale representing the frequency of fatigue perceived by respondents, with scores from 1 to 5 corresponding to a frequency of never (0%), rarely (25%), sometimes (50%), often (75%), and always (100%), respectively. Higher scores indicate greater job burnout, and vice versa. The Cronbach’s α for the four dimensions ranged from 0.84 to 0.92 for both male and female participants, demonstrating high reliability. Additionally, content validity was supported by expert review and factor analysis, which revealed a cumulative explained variance of approximately 67%. To further ensure measurement rigor, CFA was conducted in this study, and all factor loadings exceeded 0.60, indicating acceptable construct representation. For the current sample, item 21 was removed based on reliability analysis. Factor analysis further suggested integrating personal and work-related burnout into a single dimension named "Personal Burnout" due to high conceptual overlap in the current cohort. The refined three-factor scale (Personal Burnout, Service-user Burnout, and Overcommitment) yielded an overall Cronbach’s α of 0.954, with subscale α coefficients between 0.883 and 0.914. Second-order CFA results for the modified model showed a high level of reliability and validity, with a Composite Reliability (CR) of 0.841(exceeding the 0.70 threshold) and an Average Variance Extracted (AVE) of 0.645(exceeding the 0.50 threshold). The model fit indices were excellent: χ2/df = 1.453, GFI = 0.967, AGFI = 0.943, and RMSEA = 0.042. Discriminant validity was also confirmed, suggesting that the four dimensions capture distinct aspects of job burnout.

#### Psychological capital scale

In this study, the “Chinese Version of the Psychological Capital Scale” developed by Hu et al. [86] was employed. The scale comprises 24 items, distributed across four dimensions: self-efficacy (six items), hope (six items), resilience (six items), and optimism (six items). Responses are rated on a 5-point Likert scale, from 1 “strongly disagree” to 5 “strongly agree.” Higher total scores represent higher levels of psychological capital and vice versa. The Cronbach’s α coefficients for all subdimensions exceeded 0.70, indicating good internal consistency. Confirmatory factor analysis (CFA) demonstrated that all standardized factor loadings ranged from 0.60 to 0.89, and all *t-*values reached the 0.01 significance level. For the current sample, items 20 and 23 were removed following item and reliability analyses o ensure optimal measurement quality. The final refined subscales demonstrated high internal consistency, with Cronbach’s *α* coefficients for all dimensions exceeding 0.85 and an overall *α* of 0.955. To address measurement rigor, a second-order CFA was performed to verify the structural hierarchy of the construct. After systematic model refinement based on modification indices and item loadings, the final model retained its four-dimensional structure. The standardized factor loadings for the second-order constructs were substantial ranging from 0.812 to 0.987, yielding a Composite Reliability (CR) (exceeding the 0.70 threshold) and an Average Variance Extracted (AVE) of 0.840(exceeding the 0.70 threshold). The fit indices confirmed a superior model fit: χ^2^/df = 1.492, GFI = 0.955, AGFI = 0.931, and RMSEA = 0.043. Additionally, the 95% confidence intervals of the correlation coefficients between two dimensions did not include 1 or −1, hereby providing strong empirical evidence for both convergent and discriminant validity. These results indicate that the Psychological Capital Scale is a robust measure for assessing personal resources within the JD-R and COR frameworks.

### Data processing and analysis

**Fig. 1:**
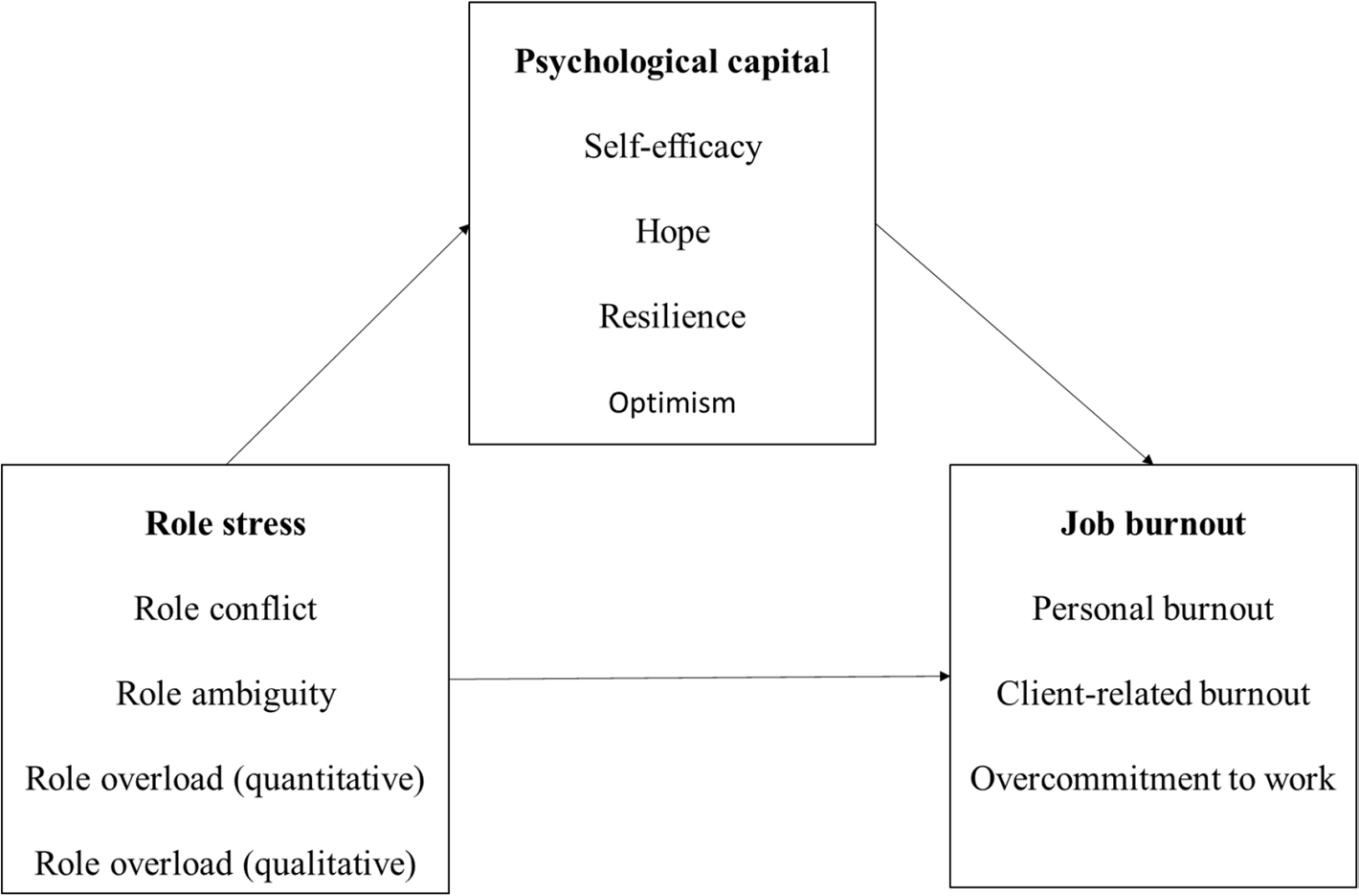
Research framework

SPSS Amos was utilized in the study to establish a structural equation model (SEM), path analysis, and CFA. This entails establishing the research framework, as illustrated in Fig. 1, followed by conducting parameter estimation and evaluating and revising the SEM based on model fit indices, concluded by testing research hypotheses via the analyses of direct, indirect, and total effects among variables. To address the potential for Common Method Bias (CMB) inherent in self-report and cross-sectional designs, several procedural and statistical remedies were implemented. Procedurally, participants were assured of their anonymity and the confidentiality of their responses to reduce social desirability bias. Statistically, Harman’s single-factor test was performed using exploratory factor analysis.

## Results

The data were collected from a single source—self-reported surveys; therefore, Harman’s single-factor test was conducted post hoc to examine CMV. The results indicated that the unrotated factor solution yielded more than one factor, and the largest factor accounted for 33.243% of the total variance, well below the 50% threshold. To further verify these results, we compared our hypothesized multi-factor model with a single-factor model using CFA. While the measurement model achieved superior fit (χ^2^/df = 2.290, RMSEA = 0.070), the single-factor model demonstrated a very poor fit (χ^2^/df = 23.627, GFI = 0.257).

This contrast confirms that a single latent factor does not explain the majority of the variance and these findings suggest that CMV was not a significant concern [85].

Before testing the structural relationships, a series of Confirmatory Factor Analyses (CFA) were conducted to evaluate the measurement model. The results indicated that the hypothesized multi-factor model achieved a superior fit (χ^2^/df = 2.290, RMSEA = 0.070, CFI = 0.908). To further address potential Common Method Bias (CMB), we compared our hypothesized multi-factor model with a single-factor model using CFA. The single-factor model yielded a very poor fit (χ^2^/df = 23.627, GFI = 0.257, CFI = 0.341), whereas our measurement model showed significantly better fit. This contrast further confirms that a single latent factor does not explain the majority of the variance, suggesting that CMB is not a pervasive issue in the current study.

### Descriptive statistics and correlation analysis

There were 262 participants in the study. Among the participants, 65 (24.8%) were male and 197 (75.2%) were female. The most common age group was 30–39 years, comprising 114 (43.5%) participants, followed by 93 (35.5%) aged 20–29 years, 41 (15.6%) aged 40–49 years, 13 (5.0%) aged 50–59 years, and one (0.4%) aged over 60 years. Regarding marital status, 161 (61.5%) participants were unmarried, 100 (38.2%) were married, and one (0.4%) was divorced. In terms of educational level, 197 (75.2%) participants held a bachelor’s degree, followed by 47 (17.9%) with a master’s degree or higher, 15 (5.7%) with an associate degree, two (0.8%) with a high school diploma, and one (0.4%) with a junior high school diploma. The most common educational background was social work or social welfare, with 86 (32.8%) participants working in this field, followed by 73 (27.9%) in nursing, 45 (17.2%) in psychology, 31 (11.8%) in counseling, 11 (4.2%) in occupational therapy, seven (2.7%) in public health, five (1.9%) in medicine, and four (1.5%) in “others” (e.g., public administration, physical therapy, sociology, and double majors in social work and criminology). A total of 91 (34.7%) participants did not hold a professional license, while the remaining 171 (65.3%) possessed one. Regarding type of the current employing organization, 112 (42.7%) participants worked in community mental health centers, 67 (25.6%) in health bureaus/departments, 50 (19.1%) in medical institutions, 16 (6.1%) in non-profit organizations or associations, 14 (5.3%) in halfway houses, and three (1.1%) in “other” institutions (e.g., health service centers). As for years of service in the current employing organization, 117 (44.7%) participants had less than three years of experience, 103 (39.3%) had three to 10 years of service, 37 (14.1%) had 10 to 20 years of service, four (1.5%) had 20 to 30 years of service, and one (0.4%) had 30 to 40 years of service. Regarding average weekly working hours, 169 (64.5%) participants worked 41–50 h per week, followed by 67 (25.6%) who worked 40 h or less, 24 (9.2%) who worked 51–60 h, and two (0.8%) who worked 61–70 h per week. See Table 1.

**Table 1:** Sample background information(*N =* 262)

Variables	Catergory	N	Percentage
Sex	Male	65	24.8%
	Female	197	75.2%
Age	20 −29	93	35.5%
	30 −39歲	114	43.5%
	40–49	41	15.6%
	50–59	13	5.0%
	60above	1	0.4%
Marriage	Single	161	61.5%
	Married	100	38.2%
	others	1	0.4%
Education level	Junior High School	1	0.4%
	Senior High School	2	0.8%
	Associate Degree	15	5.7%
	University	197	75.2%
	Graduate above	47	17.9%
Education background	Medicine	5	1.9%
	Nursing	73	27.9%
	Social work	86	32.8%
	Psychology	45	17.2%
	Counseling	31	11.8%
	Occupational therapy	11	4.2%
	Public health	7	2.7%
	others	4	1.5%
Certificate	Yes	91	34.7%
	No	171	65.3%
Types of institutions	Health Bureau/Department	67	25.6%
	Community Mental Health Center	112	42.7%
	Hospital/Clininc	50	19.1%
	Public welfare organizations/associations	16	6.1%
	Rehabilitation Home	14	5.3%
	others	3	1.1%
Years of service	Under 3yrs	117	44.7%
	3 ~ 10	103	39.3%
	10 ~ 20	37	14.1%
	20 ~ 30	4	1.5%
	30 ~ 40	1	0.4%
Average weekly working hours	Under 40 h	67	25.6%
	41 ~ 50	169	64.5%
	51 ~ 60	24	9.2%
	61 ~ 70	2	0.8%

Descriptive statistics for the levels of role stress, job burnout, and psychological capital among community mental health workers are presented in Table 1. Overall, the level of role stress among community mental health workers was at a moderate-to-low level (*M* = 2.78, *SD* = 0.78). Among different dimensions of role stress, role conflict (*M* = 3.04, *SD* = 0.979) and role overload (quantitative) (*M* = 3.08, *SD* = 1.05) were at moderate-to-high levels, while role ambiguity (*M* = 2.18, *SD* = 0.914) and role overload (qualitative) (*M* = 2.83, *SD* = 1.003) were at moderate-to-low levels. Similarly, the level of job burnout was categorized as moderate-to-low (*M* = 2.91, *SD* = 0.842). All of its subdimensions, that is, personal burnout (*M* = 2.99, *SD* = 1.032), client-related burnout (*M* = 2.78, *SD* = 0.969), and overcommitment to work (*M* = 2.94, *SD* = 1.029), were at moderate-to-low levels. By contrast, the level of psychological capital was moderate-to-high (*M* = 3.48, *SD* = 0.737). Among its four dimensions, self-efficacy (*M* = 3.63, *SD* = 0.789), hope (*M* = 3.46, *SD* = 0.852), and resilience (*M* = 3.61, *SD* = 0.788) were at moderate-to-high levels. While optimism was also at a moderate-to-high level, it exhibited a noticeably lower mean compared to the other three dimensions (*M* = 3.23, *SD* = 0.970).

The results of the correlation analysis among role stress, job burnout, and psychological capital are presented in Tables 2 and 3. Among the different dimensions of role stress, role ambiguity was weakly and positively correlated with client-related burnout and overall job burnout (*r* = 0.154, 0.223, *p <* 0.01), and weakly and negatively correlated with hope and optimism (*r* = − 0.295, − 0.220, *p <* 0.01). Additionally, role ambiguity exhibited a moderate negative correlation with self-efficacy, resilience, and overall psychological capital (*r* = − 0.354 to − 0.387, *p <* 0.01). Meanwhile, role conflict demonstrated a weak positive correlation with overcommitment to work (*r* = 0.235, *p <* 0.01) and a moderate positive correlation with personal burnout, client-related burnout, and overall job burnout (*r* = 0.342–0.379, *p <* 0.01). In contrast, it was weakly and negatively correlated with self-efficacy, optimism, and overall psychological capital (*r* = − 0.178 to − 0.236, *p <* 0.01). Role overload (qualitative) was moderately and positively correlated with job burnout and all of its subdimensions (*r* = 0.305–0.461, *p <* 0.01), while it was moderately and negatively correlated with psychological capital and all of its subdimensions (*r* = − 0.471 to − 0.394, *p <* 0.01). Similarly, role overload (quantitative) exhibited moderate positive correlations with job burnout and its subdimensions (*r* = 0.331–0.490, *p <* 0.01), whereas its correlations with psychological capital and its subdimensions were weak negative (*r* = − 0.277 to − 0.190, *p <* 0.01). Furthermore, overall role stress was moderately and positively correlated with job burnout and all of its subdimensions (*r* = 0.343–0.475, *p <* 0.01), and moderately and negatively correlated with psychological capital and all of its subdimensions (*r* = − 0.398 to − 0.302, *p <* 0.01). In sum, role overload (both quantitative and qualitative) exhibited the strongest positive correlation with job burnout. Notably, while role conflict significantly predicted burnout, role ambiguity showed a counter-intuitive negative relationship in this specific cohort, potentially due to the "diffusion of responsibility" in community mental health settings.

**Table 2:** Descriptive statistics of role stress, job burnout, and psychological capital among community mental health workers (*N =* 262)

Dimension	Mean (*M*)	Standard Deviation (*SD*)
Role ambiguity	2.18	0.914
Role conflict	3.04	0.979
Role overload (qualitative)	2.83	1.003
Role overload (quantitative)	3.08	1.050
Role stress	2.78	0.783
Personal burnout	2.99	1.032
Client-related burnout	2.78	0.969
Overcommitment to work	2.94	1.029
Job burnout	2.91	0.842
Self-efficacy	3.63	0.789
Hope	3.46	0.852
Resilience	3.61	0.788
Optimism	3.23	0.970
Psychological capital	3.48	0.737

**Table 3:** Correlation analysis among role stress, job burnout, and psychological capital

	Role ambiguity	Role conflict	Role overload (qualitative)	Role overload (quantitative)	Role stress
Personal burnout	.058	.372^**^	.431^**^	.471^**^	.429^**^
Client-related burnout	.223^**^	.342^**^	.418^**^	.331^**^	.417^**^
Overcommitment to work	.110	.235^**^	.305^**^	.419^**^	.343^**^
Job burnout	.154^*^	.379^**^	.461^**^	.490^**^	.475^**^
Self-efficacy	-.387^**^	-.200^**^	-.407^**^	-.190^**^	-.370^**^
Hope	-.295^**^	-.070	-.394^**^	-.201^**^	-.302^**^
Resilience	-.354^**^	-.100	-.401^**^	-.224^**^	-.338^**^
Optimism	-.220^**^	-.236^**^	-.431^**^	-.277^**^	-.369^**^
Psychological capital	-.356^**^	-.178^**^	-.471^**^	-.260^**^	-.398^**^

^**^p < .01, *p < *.05*

The results of the correlation analysis between job burnout and psychological capital are presented in Table 4. Among the dimensions of job burnout, personal burnout showed a moderate negative correlation with all subdimensions of psychological capital (*r* = − 0.469 to − 0.402, *p <* 0.01) and a strong negative correlation with overall psychological capital (*r* = − 0.500, *p <* 0.01). Similarly, client-related burnout was moderately and negatively correlated with all subdimensions of psychological capital (*r* = − 0.470 to − 0.417, *p <* 0.01) and strongly and negatively correlated with overall psychological capital (*r* = − 0.509, *p <* 0.01). Meanwhile, overcommitment to work demonstrated a weak negative correlation with self-efficacy (*r* = − 0.290, *p <* 0.01) and moderate negative correlations with hope, resilience, optimism, and overall psychological capital (*r* = − 0.326 to − 0.395, *p <* 0.01). Finally, overall job burnout was moderately and negatively correlated with self-efficacy, hope, and resilience (*r* = − 0.463 to − 0.492, *p <* 0.01), while its correlations with optimism and overall psychological capital were strongly negative (*r* = − 0.515, − 0.560, *p <* 0.01).

**Table 4:** Correlation analysis between job burnout and psychological capital

	Personal burnout	Client-related burnout	Overcommitment to work	Job burnout
Self-efficacy	-.402^**^	-.470^**^	-.290^**^	-.463^**^
Hope	-.446^**^	-.449^**^	-.339^**^	-.492^**^
Resilience	-.412^**^	-.435^**^	-.326^**^	-.468^**^
Optimism	-.469^**^	-.417^**^	-.401^**^	-.515^**^
Psychological capital	-.500^**^	-.509^**^	-.395^**^	-.560^**^

^**^p < *.01*

### Parameter estimation and model fit of the SEM

Model parameter estimation was conducted to test the hypothesized causal relationships. In the measurement model, the standardized regression coefficients of the latent variables on the observed variables represent the standardized factor loadings, while the squared standardized factor loadings (R^2^) indicate the proportion of variance in each observed variable explained by its corresponding latent variable. The squared multiple correlations (SMC, R^2^) are considered acceptable when they exceed 0.20 (with values above 0.50 regarded as ideal). These indices reflect the strength of association and explanatory power between latent and observed variables. Similarly, a factor loading above 0.60 is generally deemed acceptable, and one exceeding 0.70 is considered ideal, indicating satisfactory indicator reliability for the observed measures of the latent construct [87]. In our study, SMC values ranged from 0.344 to 0.883, and all factor loadings exceeded 0.60., supporting the adequacy of the measurement model.

In addition, to ensure the internal consistency and convergent validity of the constructs, composite reliability (CR) and average variance extracted (AVE) were examined. For all latent variables, the CR values (ranging from 0.841 to 0.954) exceeded the recommended threshold of 0.70, and AVE values (ranging from 0.622 to 0.840) were above 0.50, indicating satisfactory construct reliability and convergent validity. To further ensure the robustness of the measurement model, a common method bias (CMB) test was conducted using Harman’s single-factor approach. The results indicated that no single factor accounted for the majority of variance, suggesting that CMB was not a serious concern in this study.

To assess the degree of consistency between the hypothesized model and the sample covariance matrix, overall model fit indices were computed. Prior to the structural SEM analysis, a comprehensive Confirmatory Factor Analysis (CFA) was conducted to validate the measurement model and ensure construct integrity. Following conventional SEM evaluation criteria [88], multiple categories of fit indices were examined, including absolute, incremental, and parsimonious fit indices, to provide a comprehensive evaluation of model fit. Achieving a robust model fit indicates that the estimated parameters are statistically meaningful and that the model demonstrates strong empirical adequacy or the observed data. The CFA results confirmed that the measurement model achieved an acceptable level of fit, providing a rigorous foundation for testing the hypothesized structural relationships.

**Fig. 2:**
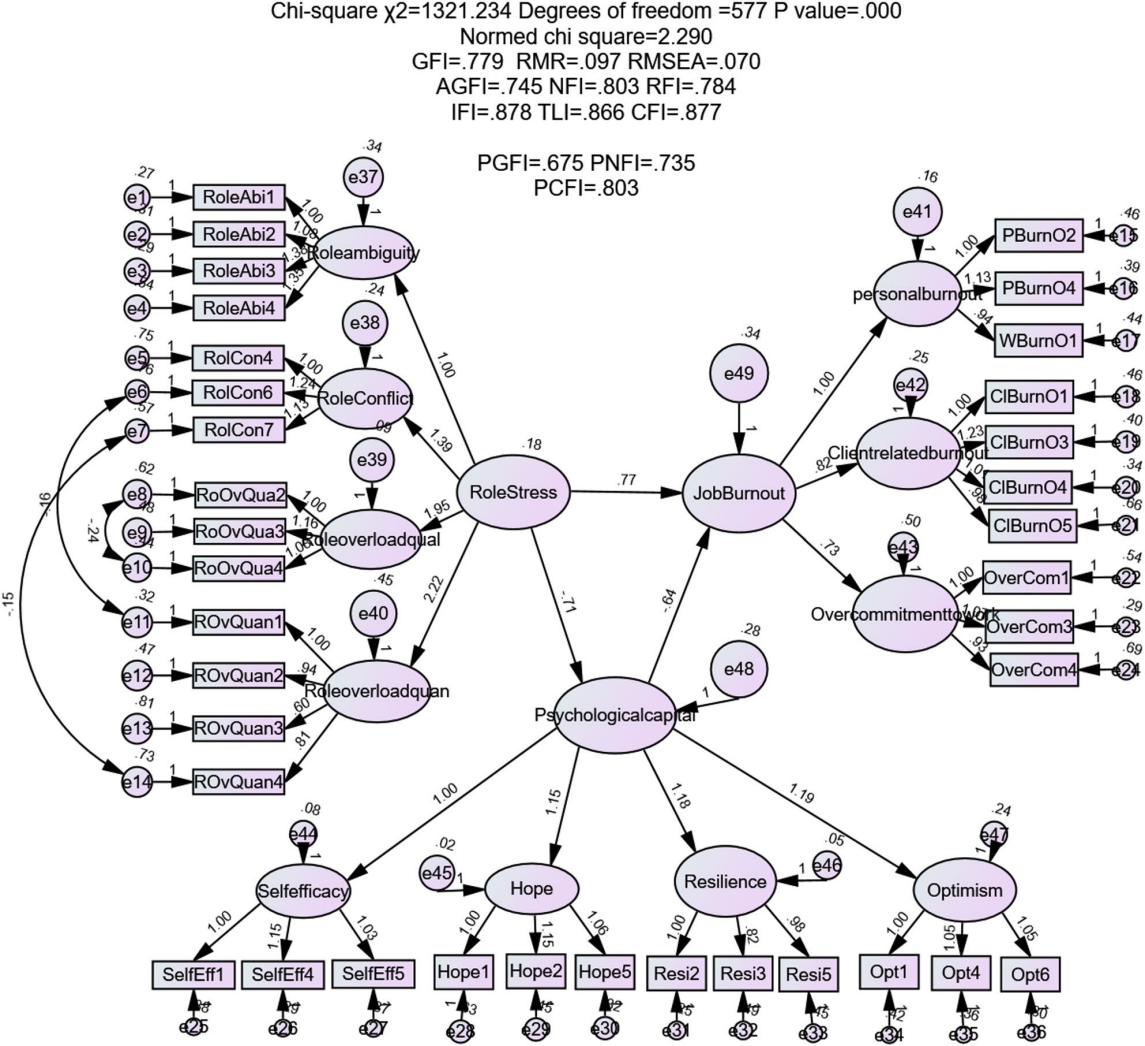
Structural equation model of role stress, job burnout, and psychological capital

The results suggest that for the proposed SEM, (1) Absolute fit indices: χ^2^ = 1321.234 (*p <* 0.001), χ^2^/df = 2.290, GFI = 0.779, and AGFI = 0.745, all of which approach the recommended threshold of 0.80, indicating an acceptable model fit. Notably, the Root Mean Square Residual (RMR) was 0.097, which slightly exceeded the recommended cut-off of < 0.08. However, the Root Mean Square Error of Approximation (RMSEA) was 0.070, which is below the 0.08 criterion and satisfies the standard for a reasonable fit [89], (2) Incremental fit indices: NFI = 0.803, NNFI = 0.866, CFI = 0.877, RFI = 0.784, and IFI = 0.878. Although these indices did not meet the recommended cutoff of 0.90, they are comparable to values reported in similar field-based studies involving complex models and real-world samples, and most were within an acceptable proximity to the recommended range; most were above 0.80 and within an acceptable proximity to the recommended range for complex models in social sciences; and (3) Parsimonious fit indices: PNFI = 0.735, PGFI = 0.675, and PCFI = 0.803. All indices exceeded the 0.50 threshold, meeting recommended standards. Furthermore, to address potential Common Method Bias (CMB), Harman’s single-factor test was performed, revealing that the first factor accounted for less than 40% of the total variance, suggesting that CMB did not significantly distort the results. Collectively, these findings indicate that, although some fit indices did not fully reach the most stringent criteria, the overall pattern demonstrates an acceptable and theoretically meaningful fit between the proposed model and the observed data, particularly given the complexity of the model and the applied research context. Figure 2 depicts the SEM framework.

### Analysis of direct and indirect effects among variables

To provide a more granular understanding of role stress, this study further examined the differential effects of its subdimensions on job burnout. Table 5 presents the results of path analysis conducted using the SEM. Among mental health workers, role stress exhibited a significant positive effect on job burnout (*β* = 0.379, *t* = 4.632, *p <* 0.001). This indicates that the higher the level of role stress experienced by community mental health workers, the greater the degree of job burnout; thus, Hypothesis 1 is supported—that is, role stress among community mental health workers has a predictive effect on their job burnout. Additionally, role stress exerted a significant negative effect on psychological capital (*β* = − 0.496, *t* = − 5.584, *p <* 0.001), while psychological capital had a significant negative effect on job burnout (*β* = − 0.457, *t* = − 5.857, *p <* 0.001). These results are consistent with the hypothesized mediation mechanism, indicating that psychological capital functions as an intervening personal resource linking role stress to burnout. These findings align with the Job Demands–Resources (JD-R) framework, where role stress acts as a demanding factor leading to health impairment, and psychological capital serves as a personal resource that mitigates burnout.

**Table 5:** Verification of the SEM for role stress, job burnout, and psychological capital

Hypothesis	Path	Standardized coefficient	t-value	Result
H1	Role stress → Job burnout	.379^*^	4.632	Supported
	Role stress → Psychological capital	-.496^*^	−5.584	
	Psychological capital → Job burnout	-.457^*^	−5.857	

^*^p <.001, indicating significance at the 0.1% level

To further examine the mediating effect, a bootstrapping procedure with 2,000 resamples was conducted, following the recommendations of MacKinnon et al. [90]. The results indicated that the indirect effect of the path “Role stress → Psychological capital → Job burnout” was 0.227 The 95% confidence interval (Bias-Corrected: 0.139 to 0.325) did not include zero (*p*-value *p <* 0.05), confirming a significant mediating role. This confirms that the indirect effect is statistically significant.

Meanwhile, the direct effect of “Role stress → Job burnout” was the direct effect of “Role stress → Job burnout” remained significant (*β* = 0.379, 95% CI: 0.201 to 0.552) indicating a significant direct effect. Additionally, the total effect of “Role Stress → Job Burnout” was 0.606, with a 95% confidence interval also excluding zero and a *p*-value less than 0.05(95% CI: 0.449 to 0.743), confirming a significant total effect.

Taken together, these findings indicate that psychological capital partially mediates the relationship between role stress and job burnout. Since both direct and indirect effects were significant and the intervals excluded zero, psychological capital was found to exert a partial mediation effect. The mediation analysis is summarized in Table 6. Collectively, these results support Hypothesis 2—that is, psychological capital mediates the relationship between role stress and job burnout among community mental health workers. This finding highlights the significant mediating role of psychological capital between role stress and job burnout. Overall, these findings showed that psychological capital serves as a mediator in the relationship between role stress and job burnout, and this mediating effect is a partial effect.

**Table 6:** Summary of the mediating effect of psychological capital

Estimate		95% Confidence Interval		
		BC/PC *p*-value	BC	PC
Indirect effect				
Role stress → Psychological capital → Job burnout	.227	.000/.001	.139 ~.325	.128 ~.318
Direct effect				
Role stress → Psychological capital	-.496	.001/.001	-.616 ~ -.350	-.620 ~ -.354
Role stress → Job burnout	.379	.001/.001	.201 ~.552	.208 ~.559
Psychological capital → Job burnout	-.457	.001/.001	-.616 ~ -.274	-.615 ~ -.272
Total effect				
Role stress → Job burnout	.606	.001/.001	.449 ~.743	.451 ~.744

*BC* Bias-corrected percentile method, *PC* Percentile method

## Discussion and conclusion

### Role Stress exhibits a positive effect on job burnout among community mental health workers

Previous studies indicated that role stress significantly exacerbates the level of job burnout among healthcare workers predominantly through role conflict [91, 92]. Care workers experiencing role conflict tend to lose their sense of professional autonomy and decision-making ability, which renders them less capable of developing predictable intervention strategies tailored to the needs of their service users, thereby leading to increased feelings of burnout. The findings of our study suggested that role stress exhibits a significant positive effect on job burnout among community mental health workers, consistent with the results of previous studies that demonstrated a pronounced positive relationship between role stress and job burnout [17, 19, 68, 70]. This consistency suggests that the detrimental impact of role stress on burnout may be robust across different healthcare systems and cultural contexts, highlighting the generalizability of this relationship.

In alignment with the Job Demands–Resources (JD-R) model, these role stressors function as critical job demands that initiate a health impairment process [13]. When workers are consistently exposed to high demands without adequate recovery, their mental and physical energy becomes depleted, directly resulting in burnout. Further regression analysis examining the relationship between different dimensions of role stress and job burnout revealed that, except for role conflict, all other subdimensions of role stress were significantly associated with job burnout. Notably, role overload (qualitative) and role overload (quantitative) showed the strongest correlations. This finding aligns with prior research emphasizing workload as one of the most critical predictors of burnout, particularly in community-based and resource-constrained settings [93]. This underscores that for Taiwan's community mental health workers, the sheer volume and complexity of tasks (quantitative and qualitative overload) are more taxing than conflicting expectations. Additionally, while overall role stress positively predicted job burnout, role ambiguity negatively predicted job burnout—a finding that contradicts the results of previous studies [93]. Such inconsistency may reflect contextual differences in organizational structure and task allocation, suggesting that the effects of role ambiguity may vary depending on how responsibilities are distributed within specific work environments.

This can potentially be attributed to the diffusion of responsibility phenomenon, according to which, individuals are more likely to exhibit proactive engagement with the assigned task when they bear the responsibility alone. Conversely, when responsibility is shared among a group, each member’s sense of accountability tends to diminish, thereby resulting in withdrawal behavior [11]. Within the specific context of Taiwan’s community mental health system, where caseloads are extremely high, unclear role expectations may sometimes reduce perceived pressure rather than intensify it, particularly in loosely structured service systems. Consequently, when the level of role ambiguity increases, that is, work tasks and responsibilities are vague, the boundaries of individual duties become more obscure.As tasks and duties are less likely to be explicitly undertaken by a single worker, each individual’s perceived sense of responsibility reduces, which, in turn, diminishes the degree of job burnout experienced. This finding suggests that the impact of role ambiguity may be context-dependent, and under certain organizational conditions, it may reduce perceived accountability and psychological pressure rather than intensify strain. This finding extends prior research by suggesting that role ambiguity may not uniformly function as a stressor but may operate differently depending on contextual and cultural factors. This suggests that in highly pressurized environments like Taiwan's mental health system, vague role boundaries might unintentionally serve as a temporary, albeit maladaptive, psychological shield against the immediate pressure of accountability. This finding extends prior research by demonstrating that role ambiguity does not uniformly function as a stressor, but may operate differently depending on systemic and cultural factors. Furthermore, by conducting a more granular analysis of role stress dimensions, these findings extend prior research by demonstrating that different dimensions of role stress do not uniformly contribute to burnout, highlighting the importance of examining role stress at a more nuanced level to better understand the complex psychological mechanisms underlying worker well-being in high-pressure service environments.

### Psychological capital exerts a partial mediating effect on the relationship between role stress and job burnout among community mental health workers

The findings of our study revealed that psychological capital exerts a mediating effect on the relationship between role stress and job burnout among community mental health workers. In other words, the relationship between role stress and job burnout in this cohort is influenced by their psychological capital. This result was consistent with prior empirical studies demonstrating the protective role of psychological capital in high-stress occupations [94, 95], and further supports its function as a key mechanism linking workplace stressors and employee well-being.

This finding can be interpreted within the framework of the JD-R model and the COR theory. The JD-R model posits that job burnout is predominantly induced by an imbalance between job demands and job resources [31, 80]. Additionally, personal resources that constitute core self-evaluations, such as self-efficacy, self-esteem, and optimism, should be considered in parallel with job resources [96]. Consistent with this perspective, previous studies have shown that personal resources not only buffer the negative effects of job demands but also promote engagement and adaptive coping [93]. Our results further validate that psychological capital acts as a vital personal resource that can buffer the negative impact of high job demands (role stress) on burnout.

In this study, role stress is perceived as the experience of inconsistent role expectations or role overload encountered by community mental health workers in response to demands from themselves, their clients, or their organizations. When such stress prevents individuals from fulfilling professional norms or institutional requirements, and when cognitive or behavioral efforts fail to alleviate the pressure or facilitate meeting expectations, role stress can be viewed as representing job demands within the JD-R framework. Conversely, psychological capital constitutes a form of personal resources. Xanthopoulou et al. [96] indicated that personal resources and psychological capital share similar contents, encompassing dimensions such as self-efficacy and optimism, while the resulting outcome is job burnout. Our findings further resonate with recent research indicating that psychological capital not only reduces burnout but also enhances professional quality of life by fostering compassion satisfaction [6]. This suggests that workers with higher psychological capital can reframe role-related challenges as opportunities for professional growth rather than mere resource drains. Therefore, when the role stress experienced by community mental health workers outweighs their psychological capital, job burnout is likely to occur. Accordingly, psychological capital can be viewed as exerting a certain degree of mediating effect on the relationship between role stress and job burnout.

The COR theory indicates that individuals strive to acquire, maintain, and protect resources they value, such as materials, social relationships, and emotional support. Stress occurs when they fear the loss of these resources, actually lose them, or fail to regain them, while burnout occurs upon experiencing a repetitive cycle of resource depletion over time [97, 98]. The theory also asserts that resources are interrelated rather than independent and can be nurtured or sustained under favorable environmental conditions [97]. This perspective is supported by recent research suggesting that resource caravans, or clusters of interrelated resources, play a crucial role in sustaining employee well-being under high-demand conditions. From this perspective, psychological capital serves as a "resource caravan" that prevents the "loss spiral" typically triggered by excessive role stress.

Community mental health workers require highly specialized professional competencies and deliver individualized services. Their professional autonomy in practice can thus be regarded as a key personal resource. However, when certain dimensions of role stress arise, such as role conflict and role ambiguity, their ability to make autonomous decisions may become constrained because of competing influences from themselves, clients, or institutions, resulting in uncertainty and internal conflict that threaten their sense of professional autonomy. Similar patterns have been observed in prior studies on human service professionals, where reduced autonomy is associated with increased emotional exhaustion and disengagement [99].

We identified that, as an intrinsic personal resource, psychological capital effectively mitigates further resource loss [100].This finding aligns with recent evidence from Kaplan and Kul Uçtu [101], who demonstrated that enhancing internal psychological strengths—such as self-esteem and communication skills—significantly improves the professional quality of life for care personnel. This finding is consistent with the core tenets of Conservation of Resources theory, which posit that individuals with greater resource reserves are better equipped to resist resource loss and recover from stress. Extending this perspective, recent studies have underscored that internal psychological strengths are particularly critical when external organizational support is limited. Within this framework, psychological capital (PsyCap) functions as a “resource caravan,” enabling healthcare professionals to mobilize and sustain personal resources, thereby enhancing their capacity to cope with and adapt to workplace stressors. While demands predict burnout, resilience and optimism act as vital buffers to maintain engagement. Both Chen et al. [102] and Chaves-Montero et al., [103] emphasize PsyCap's role in preventing health impairment. Conversely, personnel with low psychological capital who experience high levels of role stress and diminished mastery of their professional roles are more susceptible to adverse outcomes such as job burnout and even intention to withdraw. This aligns with recent longitudinal evidence indicating that insufficient psychological resources accelerate the progression from stress exposure to burnout [104].

In summary, the findings of this study suggested that psychological capital mediates the relationship between role stress and job burnout, and that psychological capital is significantly and negatively associated with both role stress and job burnout. This study makes several pivotal theoretical contributions by integrating the Job Demands–Resources (JD-R) model and Conservation of Resources (COR) theory. It extends the JD-R model by incorporating PsyCap as a critical personal resource that buffers the "health impairment process" triggered by role-related job demands. Second, it contributes to COR theory by empirically demonstrating how PsyCap functions as a "resource caravan" that mitigates the resource depletion process. By focusing on community mental health workers—a high-demand yet understudied population—this research enhances the ecological validity of these frameworks. Finally, our integrative mediation model provides a theoretically grounded "punch" by clarifying the precise explanatory mechanism linking role stress, PsyCap, and burnout.

To address burnout, institutions should develop psychological capital (PsyCap) training using international models.

We recommend that relevant institutions and training organizations develop psychological capital development training based on international models [105]. Additionally, practical strategies- including constructive feedback, manageable task assignment, and mentorship—are essential to fostering an optimistic mindset among workers. From a policy perspective, systemic changes are required to ensure sustainable caseload standards and improved resource allocation. Implementing cross-sector collaboration and integrating PsyCap into professional development will enhance workforce resilience and service quality.

While this study employed convenience sampling via an online questionnaire and adopted a cross-sectional quantitative design. To address the potential for Common Method Variance (CMV) and social desirability bias, future studies should adopt longitudinal designs or qualitative approaches, such as in-depth interviews, at the research design stage. Such nuanced exploration is vital for providing robust insights to policymakers. Ultimately, these multi-level interventions are critical for the long-term sustainability of community mental health services and the mitigation of occupational burnout.

## Data Availability

The data generated and analyzed during this study are available from the corresponding author upon reasonable request.
